# Methodology Advances in Vertebrate Age Estimation

**DOI:** 10.3390/ani14020343

**Published:** 2024-01-22

**Authors:** Yifei Zhang, Jinping Bi, Yao Ning, Jiang Feng

**Affiliations:** 1 College of Life Science, Jilin Agricultural University, Changchun 130118, China; zhang1f1998@163.com (Y.Z.); 20220811@mails.jlau.edu.cn (J.B.); 2 Jilin Provincial International Cooperation Key Laboratory for Biological Control of Agricultural Pests, Changchun 130118, China; 3 Jilin Provincial Key Laboratory of Animal Resource Conservation and Utilization, Northeast Normal University, Changchun 130117, China; 4 Key Laboratory of Vegetation Ecology of Education Ministry, Institute of Grassland Science, Northeast Normal University, Changchun 130024, China

**Keywords:** age estimation, vertebrates, methodology, accuracy assessment, conservation biology

## Abstract

**Simple Summary:**

This review introduces different methods for estimating the age of vertebrates for professionals in related fields and evaluates the accuracy of each method. Firstly, the degree of damage to vertebrates at the time of sample collection is classified. Then, the sample types and their applications are introduced, the most commonly used methods are listed, and methodological recommendations regarding accuracy are presented. Finally, the methods that will be used for vertebrate age estimation in the future, taking into account the current level of research and requirements, are predicted. This review is essential for promoting animal conservation and guiding the successful implementation of conservation management plans.

**Abstract:**

Age is a core metric in vertebrate management, and the correct estimation of the age of an individual plays a principal role in comprehending animal behavior, identifying genealogical information, and assessing the potential reproductive capacity of populations. Vertebrates have a vertebral column and a distinct head containing a developed brain; they have played an important role in the study of biological evolution. However, biological age estimations constantly exhibit large deviations due to the diversity of vertebrate taxon species, sample types, and determination methods. To systematically and comprehensively understand age estimation methods in different situations, we classify the degree of damage to vertebrates during sample collection, present the sample types and their applications, list commonly applied methods, present methodological recommendations based on the combination of accuracy and implementability, and, finally, predict future methods for vertebrate age assessments, taking into account the current level of research and requirements. Through comprehensive data gathering and compilation, this work serves as an introduction and summary for those who are eager to catch up on related fields and facilitates the rapid and accurate selection of an evaluation method for researchers engaged in related research. This is essential to promote animal conservation and guide the smooth implementation of conservation management plans.

## 1. Introduction

Due to global warming, rapid economic development, and the expansion of land utilization, nearly 28% of the 150,388 species on the International Union for Conservation of Nature (IUCN) Red List are classified as endangered. Species conservation is widely recognized as a crucial issue for humans, and biologists are increasingly researching the ages of animals, as it has serious effects on mortality, parasite susceptibility, and reproductive life history [1,2,3,4]. While a plethora of techniques exist for ascertaining the age of animals, ethical issues may arise in the process of selecting samples, and the precision of the approach is subject to variation contingent upon the sample and species, leading to confusion among researchers.

Some of these approaches have become widely used, such as skeletochronology [5], asymptotic models [6], near-infrared spectroscopy measurements [7], and DNA methylation [8]. Furthermore, age estimation accuracy is heavily influenced by the sample type. Depending on the level of interference with and injury to animals during collection, sampling techniques can be broadly classified into three categories: destructive sampling, non-destructive sampling, and non-invasive sampling [9]. Destructive sampling refers to the process of slaughtering animals to obtain specimens such as otoliths, bones, and eyeballs. Non-destructive sampling involves capturing animals to obtain blood, teeth, scales, or other materials for analysis. However, this method may subject the animals to varying degrees of pain, and the use of this method for rare or endangered species may be restricted due to ethical concerns [9,10]. Meanwhile, non-invasive sampling is broadly employed in research projects, particularly in wildlife studies where animals are not subjected to physical interventions. The samples collected may include feces, urine, vocalizations, or hair [11,12]. With the advancements in research technology, an increasing number of sample types and methods are being employed for animal age estimations.

Due to the limitations of the sample types or methodologies, extrapolated values can significantly deviate from the actual age of animals, whereas for economic animals, biological age serves as the foundation for determining the quantity and timing of harvest to prevent over-exploitation and industry collapse. As exemplified by the horsemouth bass (*Theragra chalcogramma pallas*), incorrect assessments of historical age have resulted in erroneous population biomass evaluations, reducing production by over 100-fold in less than a decade [13,14,15]. Furthermore, the obtained animal age information aids in studying the age structure and several essential life history parameters of endangered animals. Subsequently, appropriate conservation measures and management policies can be implemented according to the population’s survival status to promote steady and sustainable growth.

In this review, we comprehensively study a variety of common sample types, methods, and mechanisms for age estimation, and objectively assess the strengths and limitations of each method based on animal species. This will enable us to answer three scientific questions: (1) What are the current metrics that vary with age and by what methods can they be quantified? (2) Which experimental materials are ideal for accurate assessment while adhering to research species restrictions? (3) Where are the further opportunities for breakthroughs or optimizations in this field? This paper can assist scientists to efficiently navigate the current state of research in this field, as well as guide researchers to select appropriate samples and identification methods for obtaining accurate animal age information. Ultimately, this will establish a solid research foundation for the proper management and rescue of rare wildlife and advance the discipline of conservation biology.

## 2. Destructive Samples

### 2.1. Skeletons and the Most Calcified Structures of the Body

The age of most animals can be estimated by examining the condition of their bones and the growth pattern of their skeletons [16]. Skeletochronology, a classic method that involves identifying growth layers in long bones by counting the lines of arrested growth (LAGs) [5,17,18], has been used in various vertebrates, including fish [19], amphibians [20,21], reptiles, and mammals [22,23].

The age of an individual can be estimated through an examination of calcified tissues within the body. In fish, there are vertebrae, opercular bones [24,25], and metapterygoid bones [26,27]. Vertebrae are most commonly used for measurements; the fourth or fifth vertebrae located behind the skull are extracted from an animal’s body, excess tissue is removed and dried, and then the ring on one side of the vertebral body is observed via a microscope to obtain age information. This method has been applied to the smalltooth sawfish (*Pristis ectinate*), common carp (*Cyprinus carpio*), misgurnus (*Misgurnus anguillicaudatus*), and large-scaled loaches (*Paramisgurnus dabryanus*) [28,29,30,31]. Some researchers, however, obtain bones from various parts of the body to determine mammalian age; for instance, Sanfelice et al. used the developmental status of the skulls of South American fur seal (*Arctocephalus australis*), Northern fur seal (*Callorhinus ursinus*), and South American sea lions (*Otaria byronia*) to infer their respective ages [32], and Kryštufek et al. exploited the incremental bone layers of the mandible to confirm the age information of edible dormice (*Glis glis*) [33].

### 2.2. Eyeball Lens

Previous studies have demonstrated that mammalian eyeball lenses exhibit sustained growth at varying growth rates, characterized by a rapid growth trend in early stages, followed by slower growth throughout the life cycle until approaching an asymptotic maximum. Furthermore, nutritional factors and gender do not appear to affect the growth of the eyeball lens [34,35]. However, the death of an animal leads to a decrease in lens temperature, which results in reduced activity of the ion pump, and ultimately disrupts the linear relationship between lens mass and age [36,37]. Based on this, several studies have determined the age of animals by measuring the lens’s dry weight, for example, in the red kangaroo (*Macropus rufus*), eastern grey kangaroo (*M. giganteus*) [38], gray foxes (*Urocyon cinereoagenteus*) [39,40], black-backed jackals (*Canis mesome las*) [41] and hog deer (*Axis porcinus*) [37].

Furthermore, the age of an organism can also be determined by detecting proteins in the nucleus of the eyeball lens. Living organisms biochemically produce only the L-enantiomers of amino acids, and with increasing age, the L-type amino acids of the organism gradually undergo racemization to D-type amino acids; the ratio of D-type to L-type amino acids (D/L value of amino acids) increases with age. Proteins in the nucleus of the eyeball lens are among the most stable proteins in mammals, and racemization can be detected [42,43]. Aspartate is the most commonly used amino acid in mammalian age estimation because it has the fastest rate of racemization in animal tissues [44,45]. Therefore, some studies have used the lens to estimate the age of animals using aspartic acid racemization (AAR), a method commonly used in the estimation of the age of large marine mammals, including Bowhead whales (*Balaena mysticetus*), humpback whales (*Megaptera novaeangliae*) and narwhals (*Monodon monoceros*) [46,47,48].

In addition, some studies have applied radiocarbon dating technology to estimate the age of fish by obtaining a chronology of the eye lens nucleus [49]. In vertebrates, the eye lens nucleus is composed of metabolically inert crystalline proteins, which are formed during embryonic development and retain proteins synthesized at the age of zero [50,51]. This feature has been used in some marine vertebrates whose ages are difficult to estimate through skeletal growth band counting [49], especially in many shark species like Greenland sharks (*Somniosus microcephalus*), black dogfish (*Centroscyllium fabricii*), and white sharks (*Carcharodon carcharias*) [49,51,52].

### 2.3. Otoliths

Otoliths are essential structures in the inner ear of vertebrates that are composed of a protein matrix and calcium carbonate and play a critical role in spatial orientation and auditory perception [53,54,55]. As they increase alongside the growing organism and are not absorbed, using them as a sample for age estimation yields a higher accuracy compared to other methods [30,56,57]. A commonly used method for age estimation through otoliths involves making cuts at the core of the otolith to examine concentric circles, from which the age of various fish species such as white crappie (*Pomoxis annularis*), black crappie (*Pomoxis nigromaculatus*), northern barracuda (*Albula vulpes*), and canopy fish (*Gadus chalcogrammus*) has been inferred [27,57,58,59]. Since there is a predictable positive correlation between age and otolith mass [60], the age of animals, like the black line sharks (*Labeo bata*) [61], can also be identified with the weight of the otolith.

Additionally, otoliths can be utilized for fish age determination through lead-radium dating, bomb radiocarbon (^14^C) dating, and Fourier-transform near-infrared spectroscopy. Lead-radium dating is based on the incorporation of naturally occurring radium-226 (^226^Ra) into the otolith and its decomposition to lead-210 (^210^Pb) [62]. By measuring the disequilibrium of the ^210^Pb and ^226^Ra radioisotopes in an otolith sample, an independent estimation of age can be derived based on the known growth rate (to determine ^210^Pb, perform α-spectrometry of ^210^Po, its daughter product, and determine ^226^Ra using a technique that uses isotope-dilution thermal ionization mass spectrometry [63,64,65,66]). Bomb radiocarbon dating is used for fish otoliths, the measured bomb ^14^C levels of which are compared with regional ^14^C reference records to provide an estimate of age [62,67]. An innovative method for determining fish ages through otoliths using Fourier-transform near-infrared spectroscopy and partial least squares regression models has been developed. Compared to the traditional method of counting annuli in the otolith, this approach offers substantially greater efficiency, better precision, and higher repeatability [68].

## 3. Non-Destructive Samples

### 3.1. Blood

Blood is commonly utilized as a non-destructive sample for DNA methylation (DNAm) analysis, which focuses on the methyl group of cytosine followed by guanine (CpG site). This area has been extensively researched, and recent evidence supports the use of epigenetic modification to determine an individual’s age [69,70,71]. There are two types of age-related DNAm: epigenetic drift and epigenetic clock [70]. The latter has a high generalizability and has already been applied to the age estimation of humans and other animals [8,72,73].

In addition, the physiological and biochemical indicators in blood can aid in determining the age of an animal. Hematology and serum/plasma biochemical values in ectothermic vertebrates exhibit significant inter- and intra-species variation, which can be attributed to factors such as nutrition, sex, age, population dynamics, and environmental conditions [74]. Furthermore, hematologic and serum biochemical analyses can also be used to determine the age class of animals [75]. For example, Tóthová et al. conducted a comparative analysis of serum protein concentrations and fractions (albumin, α1-, α2-, β1-, β2-, and γ-globulin) in the blood of cattle of various ages, determining that the relative concentrations of α1-, α2-, and β1-globulin in young calves were substantially higher compared with older animals [76], while Khalil et al. examined the testosterone in horse blood and found that immature horses had lower testosterone concentrations compared to mature horses [77]. Additionally, other physiological and biochemical parameters in blood, such as the heterophil to lymphocyte ratio (H/L), the red blood cell count (RBC), serum creatinine levels, and serum uric acid concentrations, can also serve as fundamental indicators for determining an animal’s age [78,79].

### 3.2. Scales

Fish scales are hard, thin, lamellar structures derived from the body surface that increase in proportion to body surface growth and have a protective effect [80,81]. For nearly a century, fish scales have served as a reliable means of documenting crucial information regarding the age and life history of fish [82]. Fish growth cycles are calculated in a similar way to otolith rings [82]; both are extrapolated from the number of “rings” in the sample. Fish scales are easily collected and observed for age identification, and their abundance makes them a regular choice for assessing the age of samples.

### 3.3. Fin Rays

Fin rays are derived from scales or scale-like structures [83] and have a growth pattern in which new layers of fins wrap around old ones, revealing a ring-like structure in the cross-section [82], which is commonly utilized for age estimation. Among various species, the excision of fin rays prompts the regeneration of osteoblasts, which eventually reconstruct and restore the function of fins [84]. Therefore, pectoral fin rays have been widely utilized in determining the age-related information of living animals [85,86].

### 3.4. Fin Spines

Fin spines have a structure that is unsegmented, more rigid, and more calcified than fin rays [82]. Fin rays are spine-like sclerites that support the fin’s membrane and are primarily composed of calcium carbonate [87]. The utilization of fin spines is advantageous for age determination in species with irregularly shaped otoliths. For some species, fin ray and spine ages are in close agreement with otolith ages, providing a non-lethal method for estimating fish age [88,89].

Fin spines are commonly used to estimate the age of sturgeon by counting the growth bands [90,91], as well as some sharks such as black dogfish (*Centroscyllium fabricii*) and spiny dogfish (*Squalus acanthias*) [51,92]. In the case of the largemouth bass (*Micropterus salmoides*), common carp (*Cyprinus carpio*), and other species, fin spines provide a more accurate means of age estimation than scales [93,94,95]. However, age estimation is challenging for many deep-water cartilaginous fish due to their poor calcification and lack of visible growth bands [96,97]. Near-infrared spectroscopy (NIRS) is a potential alternative to traditional aging methods and requires developing a calibration model in conjunction with conventional band-counting techniques. Once a calibration model has been developed, NIRS can be used to scan the dorsal fin spines that have not been aged via conventional band counting and the calibration model can be applied to estimate their age [98].

### 3.5. Teeth

Teeth can serve as non-destructive samples to determine the age of most mammals, and researchers typically use cementum annuli [99], tooth replacement and wear (TRW) [100], and the number of layers of tooth growth for this purpose [101].

Methods for the eruption of deciduous and permanent teeth are only reliable when the second dentition is over 6 months old [102]. A library of age-related tooth phenotypes has been established based on tooth wear, eruption patterns (deciduous and permanent teeth), coloration and staining, and comparison with reference samples of a known age [103]. Olifiers et al. continuously observed and quantified tooth eruption and wear patterns in brown nose hares (*Nasua nasua*) and crab-eating foxes (*Cerdocyon thous*) to construct teeth condition indices, which can be used to estimate animals of unknown age [104].

TRW is a commonly utilized method for determining the age of large mammals, particularly herbivores such as the white-tailed deer (*Odocoileus virginianus*), elk (*Elaphurus davidianus*), and red deer (*Cervus elaphus*) [100,103]. However, TRW can also serve as a reliable indicator for carnivore age estimation. For instance, Gipson et al. utilized tooth wear to estimate the age of grey wolves (*Canis lupus*) [105]. Based on tooth wear, bats can be classified into age classes; corresponding reference standards have been established in Chiroptera, including short-nosed fruit bats (*Cynopterus sphinx*), noctules (*Nyctalus noctule*), and big brown bats (*Eptesicus serotinus*) [106,107].

The age of marine mammals can be determined by counting the growth layer groups (GLGs) in their teeth. This method estimates age by tallying the incremental growth layer groups that are annually deposited in either the cementum or dentine [108]. The age of both California sea lions (*Zalophus californianus*) and harp seals (*Pagophilus groenlandicus*) can be calculated via this method [101,109]. For most species of the Cervidae family, cementum annulus analysis (CAA) is utilized for age determination. The process involves extracting the tooth roots, typically incisors, sectioning and staining them, and examining them under a microscope to count the annuli of the teeth, each representing one year of age [100]. This method has been used for some hoofed animals, like moose (*Alces alces*), mule deer (*Odocoileus hemionus*), spotted deer (*Axis axis*) [99,103,110,111], and some chiropteran animals too, like flying foxes (*Pteropus lecto*), grey-headed flying foxes (*Pteropus poliocephalus*), and short-nosed fruit bats [107,112]. This method is applicable for age estimations for a limited number of carnivorous mammals, such as red foxes (*Vulpes vulpes*) and tigers (*Panthera tigris*) [113,114]. However, its implementation in the field is complicated due to the requirement for laboratory-based extraction and analysis of teeth. Additionally, ethical concerns may arise [115].

Gum-line recession is a widely used parameter for estimating age in felines and involves measuring the distance between the juvenile and its current gumline. This technique has been employed to estimate age in various species, including mountain lions (*Puma concolor*), lynxes (*Lynx canadensis*), and tigers [114,116,117].

Due to the formation of a new dentin layer in the pulp cavity during the growth of mammalian teeth, as individuals age, the size of the pulp cavity decreases. Therefore, the size of the pulp cavity can potentially indicate animal age [118]. Baagøe developed a technique in 1977 which involves placing canines in glycerol to measure the width of the pulp cavity using an ocular micrometer under a dissecting microscope [118]. The ages of both noctules and big brown bats have been estimated by this method [106].

## 4. Non-Invasive Samples

### 4.1. Feces

Animal feces primarily comprise food remnants, microorganisms, gastrointestinal secretions and debris, metabolic hormones, water, and minerals. This sample is a common, non-invasive method to obtain basic information about animals. Some studies have shown that the morphology of feces and their microorganisms can be used to estimate the age and health status of wildlife [119,120,121]. For instance, Rueda et al. concluded that the pellet size of European hare (*Lepus europaeus*) feces is directly proportional to their age [122]. Kongrit et al. also discovered that the circumference of wild Asian elephant (*Elephas maximus*) feces can be used as a reliable indicator to estimate their age [123]. Furthermore, Wiedower et al. used fecal near-infrared reflectance spectroscopy (FNIRS) to discriminate between different age classes of giant pandas (*Ailuropoda melanoleuca*); they grouped feces from known age classes, and after obtaining fecal spectral data, with one part used for calibration and the other for validation, they made inferences about the animals’ age classes [7].

### 4.2. Physical Characteristics

Growth is a crucial parameter that characterizes the life history of individuals or a species within an ecological context [124,125,126,127]. It is consistently associated with natural mortality [128,129], size-based survival [130], life span [131], and expected abundance [132]. In the field of growth studies, the three most commonly utilized asymptotic models are the Gompertz model, Richards’ growth model, and the von Bertalanffy growth function (VBGF) model [6,130]. These models utilize growth functions to fit the age data of animals, enabling inferences about approximate age groups.

Feathers are a recognizable physical characteristic of birds, and are crucial for their flight, self-defense, and reproduction. Some studies have inferred the age of birds based on their molting and feather patterns, for example, olive-streaked flycatchers (*Mionectes olivaceus*), American dippers (*Cinclus mexicanus*) and house wrens (*Troglodytes aedon*) [133,134,135].

Trunk or limb parameters can be used to estimate the age of certain animals. For instance, in Western grey kangaroos (*Macropus fuliginosus ocydromus*), head, foot, and leg lengths are positively correlated with age [136]. The age of wild Asian elephants (*Elephas maximus*) can be estimated through a comparison of their shoulder width and height with the corresponding parameters of Asian elephants of a known age [137,138].

In particular, the patterns and rates of cartilage closure in some Chiropteran animals can be used as the standard for age estimation by transilluminating the wings of individuals with a headlight. Light transmission can be observed in the cartilaginous zone of the long phalanges of young individuals, while light will not be transmitted in adults due to epiphyseal plate closure [118]. In addition, there are some methods for quantitative age estimation based on bone growth, i.e., measuring the total epiphyseal gap of an individual; most bat specialists use this method to estimate the age of bats [139,140].

There are also some animals whose age can be estimated based on other physical characteristics. For example, nose color can serve as a reliable indicator of carnivore age and has the potential to be sex-specific [141]. Van Horn et al. examined the degree to which the nose color of Andean bears (*Tremarctos ornatus*) reflected their age, concluding that the ages of adults ≥10 years old may be estimated from the proportion of their nose that is pink, with an average error of <0.01 ± 3.5 years [142]. In many species, the color of juvenile and adult fur often differs, thus, also providing an important age indicator; for example, the pelage of juvenile bats is darker than that of adult individuals [118].

### 4.3. Scute

The turtle shell is composed of a dorsal carapace and a ventral plastron [143], and the scute is a keratinized structure located on the upper layer of the surface epidermis [144]. In general, the turtle species can be identified by the shape or color of the scute on the shell. These scute surfaces have continuous grooves in the shape of concentric stripes or regular annual rings, also known as growth lines, which are formed when growth is slow or stops [16,144]. Therefore, the scute is often used as a criterion to determine the age of shell turtles. Brook et al. and Howell et al. both estimated the ages of painted turtles (*Chrysemys picta*), snapping turtles (*Chelydra serpentina*), and spotted turtles (*Clemys guttata*) by using the annual rings on the scute, respectively, and evaluated the accuracy of their estimations [145,146].

### 4.4. Voice

Animal vocalizations are increasingly being utilized to monitor wildlife populations and obtain estimates of species occurrence and abundance. Stoeger et al. estimated the age groups of African elephants (*Loxodonta africana*) based on acoustic parameters, extracted from rumbles recorded under field conditions in a South African National Park; statistical models could reach up to 95% correct classification when categorizing into two groups (infants/calves in one group vs. adults) [147]. Vaytina et al. found that in the whinchat (*Saxicola rubetra*), the size of the song repertoire had an increased effect on the age estimation of males [148].

### 4.5. Hair

Hair is a convenient sample due to its easy accessibility. By measuring hormone concentrations in hair, we can gain insights into the combined hormone secretion of animals over weeks or months and determine their endocrine status. Additionally, DNA extracted from hair follicles allows us to identify individual species and their age [149].

Hormone levels in hair can vary significantly among individuals of different species, depending on factors such as sex, age, and health status. Some studies suggest that hormone concentrations may be associated with an animal’s age or sex [150]. For instance, Schell et al. inferred that the testosterone concentration in coyote (*Canis latrans*) hair could serve as a reliable indicator for distinguishing adult male coyotes from male pups [151]. In the case of wild domestic mice (*Mus musculus domesticus*), Carlitz et al. found that the testosterone concentration in the hair increases with age [152].

Other studies have also measured the methylation status of potential age-related CpG sites using the multiplex methylation SNaPshot approach and inferred tissue age from age-dependent changes in methylation sites. These studies have demonstrated the feasibility of obtaining both human identity and age information from a single scalp hair follicle [149].

## 5. Accuracy Assessment

The selection of samples and methods for age estimation is complex, and the accuracy of estimates can be influenced by different samples (Table 1). In this study, we present a comprehensive overview of age estimation accuracy based on the choice of samples and methods according to animal class.

### 5.1. Fish

**Figure 1:**
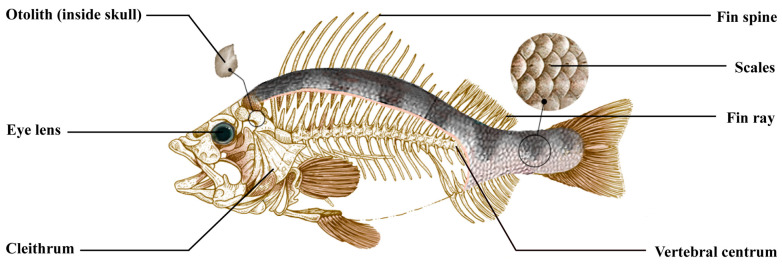
Basic anatomy of derived fishes. All alternative structures are labeled based on their anatomical locations (Cited and modified from Tzadik et al., 2017 [82]).

The parameters used to determine the age of fish are usually body length, fin rays, and the skeleton, or a variety of calcified body parts (Figure 1). The common progressive growth model for fish is the von Bertalanffy curve, which may bias the age estimation due to the large variation in individual growth rates for some species, and the fact that the positive correlation between age and body length becomes increasingly weak for elder fish. Scales are found on fish body surfaces, and the wear and tear on their surface affects the accuracy of a conclusion from their annular bands [27]. Moreover, the deceleration of scale growth with age may result in an underestimation of the age of older fish [57]. Additionally, this method of counting scales is both time-consuming and labor-intensive.

Counting the bands in the vertebrae is one of the most commonly used methods for estimating fish age. However, vertebral bands do not necessarily form every year for Chondrichthyes [96,97], so using this method may lead to underestimations of the age of elderly cartilaginous fish. In this case, radiocarbon dating can also be used to estimate individuals’ ages by using the eyeball lens [49,51,96], but both methods are destructive. Therefore, in the past 30 years, the approach of using fin spines has been most commonly utilized for estimating some cartilaginous fish ages, such as sturgeon and shark [88,92,157,158,159], and dorsal fin spines can also be used to estimate the age of some bony fish [94,95]. The combination of otolith samples with lead-radium and bomb radiocarbon dating can provide more precise age estimates for fish. However, the acquisition of otolith samples requires killing study species [62], so bomb radiocarbon dating is typically used as the preferred method for high-precision age estimations of some non-rare fish [88]. Fin sections have been used to estimate the ages of the common carp (*Cyprinus carpio*). Compared with all other structures, ages determined from pectoral fin ray sections were nearly as precise as those determined from otoliths, up to the age of 13 (lowest average percent error); thus, pectoral fin rays provide a precise, economical, and nonlethal alternative for estimating common carp ages [30].

### 5.2. Amphibia and Reptilia

For amphibians and reptiles, the most commonly used methods for age estimation are asymptotic models and skeletochronology [153]. The former method is not capable of providing an accurate estimation of an animal’s age due to inter-individual growth variations and the possibility that older animals may cease growing, leading to a misjudgment of their age [20,153]. Skeletochronology may pose a risk of harm or mortality to the animal, and some studies suggest that using this method may underestimate the age of animals. However, it remains an efficient method for obtaining the age data of amphibians and reptiles [17]. Turtles can serve as a good example; skeletochronology has been used in turtles to accurately estimate individual ages based on their humerus. However, the humerus cannot be removed from living organisms [5,17]. Therefore, in some studies, annual scute rings have been used to estimate the age of shell turtles [145]; the advantage of this method is that it is not lethal or complex, but the results obtained are not accurate enough and are not suitable for some families (Trionychidae and Dermochelyidae) [16]. Comas et al. conducted a comparative study on the efficacy of phalanges, humeri, and femurs in estimating lizard (Sauria) ages through skeletochronology, and the results revealed a strong correlation in and a high repeatability of cross-sectional readings of all bones studied [154]. Therefore, phalange skeletochronology can be utilized for age estimations in amphibians and reptiles without killing the animal. However, for limbless reptiles, i.e., snakes (Serpentes), only destructive samples (skulls) can be used to estimate age [16]. Joshua et al. mentioned that the age classes of bullsnakes (*Pituophis catenifer sayi*) can be roughly estimated based on their weight and length, but there were no further details [160].

### 5.3. Aves

The primary specimens commonly utilized for avian age determination include imagery, plumage, vocalizations, and hematology. The most widely used method for determining nestling age involves establishing appropriate aging thresholds based on observations of beak size and color and behavioral cues. The ages of Zealand fantail (*Rhipidura fuliginosa placabilis*) nestlings and European bee-eater (*Merops apiaster*) nestlings were estimated through the development of an image-based aging guide, that describes qualitative changes in appearance during their growth period [155,156]. Wails et al. proposed that feather development patterns can also serve as a reliable criterion for avian age estimation [161]. At present, the most accurate approach for age estimation in birds involves measuring biomarkers present in the blood [1].

### 5.4. Mammalia

Age estimation of marine mammals, particularly cetaceans, traditionally relied on the enumeration of growth layer groups (GLGs) present in teeth or aspartic acid racemization occurring within the eye lens [109,162]. The deposition of one GLG per year is now widely acknowledged; however, the deposition rates in other species are uncertain [163], and sperm whales (*Physeter macrocephalus*) deposit a GLG only once per year [164]. In recent years, aspartic acid racemization (AAR) in eye lenses has seen increasingly popularity for age estimation. The AAR technique can provide age estimates for all age groups of narwhals (*Monodon monoceros*) [165] but was found to be less accurate for younger individuals. This method also requires hunting the animals to remove the lens [46].

The only surviving flying mammal, bats, accounts for almost a quarter of all mammal species [166], and there are also many options for estimating their age. The age of bats can be estimated by counting incremental lines of dentine and cementum and measuring the size of the pulp cavity. However, all of these methods are invasive and require special equipment, which limits their usage and results in a low accuracy [118]. The simplest method is to observe the patterns of cartilaginous epiphyseal closure to distinguish between young bats and adults; this method only requires a headlight and can be used in both the field and laboratory [118].

For terrestrial mammals, the main samples commonly used are eye lenses, teeth, blood, feces, vocalizations, and imagery. TRW and CAA are commonly utilized for aging herbivores and carnivores [100,105]. TRW is dependent on food preferences [100]; hence, it can only be used as a reference tool for age estimation. The accuracy of cementum annuli varies geographically, with faint annuli in locations with mild winters resulting in age underestimations [100]. Additionally, ethical concerns exist [115], and for excrement, voice, and image samples, the age class of the animal can only be determined approximately with no guarantee of accuracy. Therefore, these samples are not widely utilized. Blood is the optimal sample for age estimation in terrestrial mammals due to its minimal invasiveness, and DNAm extracted from blood can accurately determine an animal’s chronological age [8]. However, DNAm also still faces some challenges: tissue specificity should be taken into account when inferring age [167,168]; certain marker DNAm patterns exhibit different manifestations at different specific ages, requiring analysis and calibration of samples at specific ages [169]; and differences caused by detection using different platforms may affect the methylation levels in the measured sites [170]. Moreover, some DNAm detection technologies have a high cost, a limited application scope, and lack universality.

## 6. The Future of Age Estimation

Current studies on animal age estimation are mainly focused on amphibians, reptiles, mammals, and especially fish, while relatively few studies are related to age estimation in birds. This may be due to the limited availability of bird samples and their aerial living environment, which pose significant challenges to sample collection and study. Age information for certain bird species can also be directly obtained through nest observations [155,156].

For terrestrial mammals, blood is the optimal sample for age estimation. Not only is it non-lethal, but DNA methylation analyses using blood samples can provide highly accurate information regarding the age of wild animals [8]. However, blood sample collection from large, wild carnivores is challenging.

The technique of utilizing the hormone concentration content (HCC) in hair samples to estimate the age class of wild animals has the potential for further refinement and application [151]. Compared to blood, obtaining hair is more convenient, and previous research has successfully inferred animal age classes based on HCCs [150,151]. Generally speaking, younger animals exhibit higher HCC concentrations, while older animals exhibit lower levels. However, it has been observed that higher HCCs occur in elderly humans and some primates [171,172]. Therefore, the age estimations in wildlife still require significant developments. Researchers must develop more accurate methods and collect valid samples to achieve this goal.

Currently, most aging studies focus on changes in the phenotype or molecular scale of organisms. However, some studies evaluate the age of organisms at the cellular level [173,174]. Cell-based technologies are used to quantify cell age, which can ultimately be used as an indicator to evaluate the cell phenotype and lifespan [175,176]. Although biases and differences may be observed between cellular senescence patterns and individual aging, the interpretation of aging at the cellular level can provide unique insights into complex life systems [176]. The value of cellular senescence in assessing biological age, cancer occurrence, and tumor suppression/promotion is very high [177]. However, cell senescence applications go far beyond this. Its potential is still waiting to be untapped by biologists.

## 7. Conclusions

In summary, we present an overview, assessment, and viewpoint of the samples and methods used to determine ages of fish, reptiles, amphibians, birds, and mammals. Individual age estimations can indicate rates of change in essential life history parameters, and age-related metrics are critical for determining the viability of endangered species populations, establishing wildlife kinship, and developing sustainable harvesting tactics. In this study, we present recommendations for obtaining animal age information, as well as references for physical applications in animal conservation and wildlife research and evaluate the accuracy of the different methods.

## Figures and Tables

**Table 1:** Commonly employed techniques, methodologies, and precision levels for age determination across various animal taxa.

Class of Animal	Structure	Lethality	Method	Study Examples	Tradeoffs
Fish	Skeleton or majority of calcified body parts	Lethal	Band counts	Blackwell et al. (2016) and Korostelev et al. (2020) [27,28]	There may be errors when counting the bands and may underestimate the age.
	Otolith	Lethal	Annuli counts	Snow et al. (2008) [58]	There may be errors when counting the annuli and may underestimate the age.
			Weight and length	Khan et al. (2018) [61]	Easy to operate, but not accurate enough.
			Near-infrared spectroscopy (NIRS)	Healy et al. (2021) [68]	Spectral analysis has a higher cost, but it has good repeatability and higher efficiency.
			Lead-radium dating	Andrews et al. (2011) [66]	Sample preprocessing needs time and effects, but it can relatively accurately obtain age information.
	Lens	Lethal	Radiocarbon dating	Nielsen et al. (2016) and Boye et al. (2020) [47,49]	Regional variability can affect the accuracy of age estimates.
	Fin rays	Non-lethal	Annuli counts	Morehouse et al. (2013) [95]	There may be errors when counting the bands and may overestimate the age.
	Fin spines	Non-lethal	Annuli counts	Hedeholm et al. (2021) [51]	There may be errors when counting the bands and may underestimate the age.
			Near-infrared spectroscopy (NIRS)	Rigby et al. (2014) [98]	Spectral analysis has a higher cost, but it has good repeatability and higher efficiency. And mostly used to evaluate deep-sea cartilaginous fishes.
	Scales	Non-lethal	Annuli counts	Ross et al. (2005) [57]	There may be errors when counting the annuli and may underestimate the age.
	Body length	Non-lethal	Growth model	Sajeevan and Kurup (2017) [6]	The standards need to be established, which requires the body length data from individuals of known ages and may underestimate the age.
Amphibia	Skeleton	Non-lethal/Lethal	Skeletochronology	Ento et al. (2002) [153]	LAGs may overlap during counting, resulting in underestimation of age.
Reptilia	Skeleton	Non-lethal/Lethal	Skeletochronology	Comas et al. (2016) [154]	LAGs may overlap during counting, resulting in underestimation of age.
	Scute	Non-lethal	Counting annual rings	Howell et al. (2018) [145]	Easy to operate, but not accurate enough.
Avia	Blood	Non-lethal	DNA methylation (DNAm)	De Paoli-Iseppi et al. (2019) [1]	It has a high cost, and not universal among different species.
	Voice	Non-lethal	-	Vaytina and Shitikov (2019) [148]	Only age classes can be judged, and not accurate enough.
	Physical characteristics	Non-lethal	-	Amiot et al. (2015) and Costa et al. (2020) [155,156]	Often used to determine the age of nestlings, and not accurate enough.
Mammalia	Skeleton	Non-lethal/Lethal	-	Kryštufek et al. (2005) [33]	Mostly used in land mammals, and it may have a large error.
	Lens	Lethal	Dry weight	McLeod et al. (2006) [38]	The standards need to be established, which requires the dry weight data from individuals of known ages.
			Aspartic acid racemization (AAR)	McLeod et al. (2006) and Boye et al. (2020) [38,51]	The samples need to be kept fresh, and the freshness of the sample may have an impact on the results.
	Blood	Non-lethal	Hematologic and serum biochemical analyses	Rørtveit et al. (2015) [75]	Easy operation, but only age classes can be judged.
			DNAm	Horvath et al. (2022) [8]	It has a high cost, and not universal among different species.
	Teeth	Non-lethal	Tooth replacement and wear (TRW)	Rosatte et al. (2007) [103]	The results may have some substantial errors.
			Growth layer groups (GLGs)	Rust et al. (2019) [101]	As age increases, the difficulty of calculating GLG often increases.
			Cementum annuli analysis (CAA)	Asmus et al. (2011) [99]	There may be errors when counting the annuli and not accurate enough.
			Gum-line recession	Fàbregas and Garcés-Narro (2014) [114]	Not accurate enough.
			Size of pulp cavity	Gol’din et al. (2018) [106]	There may be errors in elderly individuals
	Feces	Non-lethal	Size	Kongrit and Siripunkaw (2017) [123]	Easy to operate, but only age classes can be judged, and not accurate enough.
			Near-infrared spectroscopy (NIRS)	Wiedower et al. (2012) [7]	Spectral analysis has a higher cost, and only age classes can be judged.
	Hair	Non-lethal	Hormone	Cattet et al. (2018) [150]	Hormone extraction costs are relatively high, and only specific hormones have a significant correlation with age.
			DNA methylation (DNAm)	Hao et al. (2021) [149]	It has a high cost, and not universal among different species.
	Voice	Non-lethal	-	Stoeger et al. (2014) [147]	Only age classes can be judged, and not accurate enough.
	Physical characteristics	Non-lethal	-	Van Horn et al. (2015) [142]	Only age classes can be judged, and not accurate enough.

“-” indicates no fixed method.

## Data Availability

No new data were created or analyzed in this study.
